# *Salmonella* LVR01 triggers antagonistic two-armed innate immune memory that impacts on antitumor efficacy

**DOI:** 10.3389/fimmu.2025.1535131

**Published:** 2025-04-30

**Authors:** Sofía Chilibroste, Jéssica C. dos Santos, Amy Mónaco, Leo A. B. Joosten, María Moreno, José A. Chabalgoity

**Affiliations:** ^1^ Departamento de Desarrollo Biotecnológico, Instituto de Higiene, Facultad de Medicina, Universidad de la República, Montevideo, Uruguay; ^2^ Department of Internal Medicine, Radboud University Medical Center, Nijmegen, Netherlands; ^3^ Department of Medical Genetics, Iuliu Hatieganu University of Medicine and Pharmacy, Cluj-Napoca, Romania

**Keywords:** *Salmonella*, innate immune memory, trained immunity, tolerance, cancer immunotherapies, anti-tumor Immunity

## Abstract

The current understanding of innate immune memory encompasses both trained immunity and immune tolerance, where cells can exhibit enhanced responsiveness or immune paralysis upon subsequent stimuli, respectively. Various agents induce either of these responses, including β-glucan, *Leishmania*, BCG and LPS. BCG is a clinically approved immunotherapy for bladder cancer and BCG-induced trained immunity is important in driving anti-tumor adaptive immunity. *Salmonella* also shows promise in cancer treatment, eliciting potent anti-tumor immune responses, but with transitory effects. This led us to investigate whether *Salmonella* LVR01, like BCG, triggers trained immunity and its impact on anti-tumor responses. Herein, we report that *Salmonella* induces an enhanced response in bone marrow cells, characterized by a robust cytokine response upon a second stimulus, in a fashion that resembles trained immunity. Coherently with that, *Salmonella* administration induces enhanced responsiveness to a tumor implanted later in time, resulting in slow tumor growth and extended survival. However, *in vitro* stimulation of human monocytes and murine bone-marrow derived myeloid-enriched cells with *Salmonella* results in decreased production of cytokines resembling immune paralysis. Overall, our results suggest that *Salmonella* LVR01 induces enhanced responses of innate immune memory, as well as paralysis on monocytes. These two antagonistic effects could be the basis of the transitory effect of *Salmonella* treatment and suggest that further investigation on these phenomena could shed light on how to improve *Salmonella*-based immunotherapies for cancer.

## 1 Introduction

Innate immune memory has been thoroughly explored in the context of diseases, revealing various underlying mechanisms. Innate immune cells exhibit the capability for epigenetic reprogramming in response to a stimulus, resulting in increased responsiveness to subsequent stimuli, known as trained immunity, or a shift towards a state of tolerance characterized by a diminished response and immune paralysis (1–3). Lipopolysaccharide (LPS) has been established as a key inducer of tolerance, while agents like *Candida albicans*, β-glucan, *Leishmania* species lysates, and Bacillus Calmette-Guérin (BCG) have been recognized for their role in promoting trained immunity (4–6). Our interest lies in BCG because it is a live attenuated vaccine with diverse applications, serving both as an anti-tuberculosis vaccine and an immunotherapeutic agent for cancer treatment (7, 8). In our research, we employ an attenuated strain of *Salmonella* Typhimurium for cancer immunotherapy and have noted significant similarities between the application of BCG and *Salmonella* in cancer treatment scenarios. Both bacteria share the characteristic of being capable of eliciting an immune response against neoplastic cells (9–12). Initially, they activate the innate immune system by signaling through microbial-associated molecular patterns (MAMPs) and damage-associated molecular patterns (DAMPs), ensuing from the controlled initial infection (13, 14). Subsequently, they elicit an adaptive immune response directed against tumor cells, with the induction of immunogenic cell death, which provides antigens and signaling, facilitating T and B cell response (14, 15). The presence of LPS on the surface of *Salmonella*, as a difference from BCG, cannot be overlooked, given its well-established role as a potent inducer of innate immune tolerance (16). Moreover, LPS significantly contributes to the anti-tumor response by triggering the initial immune response (17). Hence, we are motivated to investigate whether *Salmonella*, analogously to BCG, can induce trained immunity and if this mechanism contributes to the anti-tumor response. *In vitro* experiments demonstrated that *Salmonella* induces immune tolerance in adherent monocytes, but not in other bone marrow-derived cells, likely attributable to robust initial stimuli. However, intriguingly, our *in vivo* investigations revealed that prophylactic administration of *Salmonella* in mice elicits a strong cytokine response throughout the bone marrow cells and confers protection against cancer upon pre-treatment. These results suggest that *Salmonella* could promote heterologous immunomodulation useful in the prevention of cancer through the modulation of the innate immune environment.

## 2 Materials and methods

### 2.1 Cell lines

B16F10 melanoma cells were purchased from ATCC (CRL-6475) and maintained in DMEM medium (Capricorn Scientific, cat. DMEM-HPSTA) supplemented with 10% fetal bovine serum (Capricorn Scientific, cat. FBS-11A) at 37 °C in 5% CO_2_ atmosphere.

A20 lymphoma cells were purchased from ATCC (TIB-208) and maintained in RPMI-1640 medium (Capricorn Scientific, cat. RPMI-STA) supplemented with 10% fetal bovine serum (Capricorn Scientific, cat. FBS-11A), 0.05 mM 2-mercaptoethanol (Sigma-Aldrich), 1 mM sodium pyruvate (Sigma-Aldrich), and additional 2.5 g/L D-glucose (Sigma-Aldrich) at 37 °C in 5% CO_2_ atmosphere.

### 2.2 Bacterial strain

*Salmonella enterica* serovar Typhimurium LVR01, an attenuated strain constructed by introducing a null deletion into the *aroC* gene of the parental canine *S.* Typhimurium isolate P228067 (18), was employed in the present study. Bacteria were routinely cultured at 37 °C in LB broth (Difco), shaken at 200 rpm overnight and stored at -80 °C in 15% glycerol stocks until needed. For *in vivo* experiments, bacteria were thawed and resuspended in saline solution to a final concentration of 1 x 10^7^ CFU/ml. The bacterial inoculum was verified through serial dilution and plating in LB agar (LBA, Difco).

### 2.3 *Leishmania* parasites

*Leishmania braziliensis* strains were obtained from Leishbank IPTSP/UFG (Goia´s, Brazil). Promastigote forms were cultured in Grace’s insect medium (Gibco, Life Technologies, USA) supplemented with 20% of heat-inactivated fetal bovine serum (FBS, Gibco, Life Technologies, USA) and 100 U/mL of penicillin/streptomycin (Sigma-Aldrich) at 26 °C. Stationary-phase parasites were obtained on the 6th day of growth and washed three times with PBS (1000 xg, 10 min 10°C). After, they were suspended in PBS and quantified by using hemocytometer after fixation with PBS/0.4% formaldehyde. Parasite lysates were obtained by 5 freeze-thaw cycles of promastigotes in the presence of protease inhibitors (Protease inhibitor cocktail, Sigma-Aldrich) in liquid nitrogen followed by thawing in a water bath at 37 °C. Protein quantification was performed by using Pierce BCA protein assay kit (ThermoFisher Scientific, USA).

### 2.4 Adherent human monocyte isolation

Monocytes were isolated from buffy coats obtained from healthy donors (Sanquin Blood Bank, Nijmegen, the Netherlands). Peripheral blood mononuclear cells (PMBCs) were isolated by differential density centrifugation over Ficoll-Paque (GE Healthcare) of blood diluted in PBS. Cells were washed three times with PBS and quantified. PMBCs were put on a hyperosmotic Percoll solution (48.5% Percoll (Sigma-Aldrich), 41.5% sterile H_2_O, 0.16 M filter-sterilized NaCl) and centrifuged for 15 min at 580 xg, 4 °C. The interphase layer was isolated, and cells were washed once with cold PBS and suspended in Dutch-modified RPMI 1640 culture medium (Invitrogen) supplemented with 5 µg/mL gentamicin (Centraform), 2 mM Glutamax (Gibco) and 1 mM pyruvate (Gibco) and counted. Finally, adherent monocytes were isolated by allowing Percoll-isolated monocytes to adhere to polystyrene flat-bottom plates (Corning) for 1 h at 37°C; the cells were washed with warm PBS to obtain maximal purity (7).

### 2.5 *In vitro* induction of trained immunity in human adherent monocytes

Cells were incubated with culture medium supplemented with 10% human pooled serum together with different stimuli: supplemented medium as a negative control, 25 μg/ml of *L. braziliensis* lysates as a positive control (5), different MOI (multiplicity of infection) of LVR01:cells (1:1, 1:2, 1:4, 1:8, 1:16, 1:32) and heat-killed (hk) LVR01. After 1 h, cells were washed with warm PBS and culture medium with gentamicin was added. After 24 h, supernatants were collected and stored at -20°C until further use. After 3 days, culture medium was refreshed. On day 6, cells were restimulated with culture medium without serum, 10 ng/mL LPS (Sigma-Aldrich) or 10 µg/mL Pam_3_Cys (Sigma-Aldrich). After 24 h, supernatants were collected and stored at -20°C until further use.

### 2.6 Mice

Female C57BL/6 and BALB/C mice (Dilave, Uruguay), 6–8 weeks old, were used for *in vivo* experiments. Animals were housed on 12:12 h light/dark cycles with controlled temperature (22 ± 2°C) and humidity (60%), with water and food *ad libitum*. All animal experimentation protocols were approved by the University’s Ethical Committee for Animal Experimentation, Uruguay (protocol number 1329).

### 2.7 *In vitro* induction of trained immunity in murine bone marrow-derived enriched myeloid cells

Fresh bone marrow cells were isolated from the femurs of C57BL/6 and BALB/C mice. Bone marrow was aseptically removed, and cells were incubated with Lysis Buffer (155 mM NH_4_Cl, 10 mM KHCO_3_ in sterile H_2_0), filtered through a 40 µm cell strainer (Corning 431750, Sigma-Aldrich), and resuspended in RPMI (Capricorn Scientific, cat. RPMI-STA) supplemented with 10% fetal bovine serum (Capricorn Scientific, cat. FBS-11A), Penicillin-Streptomycin (Capricorn Scientific, cat. PS-B) and 1 mM sodium pyruvate (Sigma-Aldrich). Cells were counted and plated in a 96 well flat bottom plate (1 x 10^6^ cells/well). During the first 24 h cells were stimulated with 50 μg/ml of L. *braziliensis* lysates (positive control), 1 x 10^6^ CFU of hk *Salmonella* LVR01, or supplemented medium (negative control). Subsequently, cells were washed with 200 µl of warm PBS to wash away the stimuli and non-adherent cells, and fresh medium was added. After 3 days, culture medium was refreshed. After six days of total rest, cells were restimulated with culture medium without serum, 10 ng/ml LPS (Invivogen) or 100 µg/ml Poly I:C (Sigma-Aldrich). After 24 h, supernatants were collected and stored at -80°C until further use.

### 2.8 *Ex vivo* evaluation of *in vivo* trained immunity model

To investigate the effect of inducing trained immunity on the bone marrow, mice were inoculated intraperitoneally (i.p) with saline or 1x10^6^ CFU of Salmonella LVR01. After 6 days, bone marrow was removed. 1 x 10^5^ bone marrow cells were cultured per well in two different plates One plate was immediately restimulated *ex vivo* with LPS (10 ng/ml), and 24 hours later, the supernatant was collected to detect cytokine levels. Besides, adherent cells were characterized by flow cytometry (**Supplementary Figure 1**). On the other plate, adherent cells derived from bone marrow were allowed to rest for 6 days and then restimulated with LPS (10 ng/ml), 24 hours after, the supernatant was collected.

For *in vivo* trained immunity immunization experiments, mice were inoculated with saline or LVR01 i.p, and 7 days later, 35 µg/dose LPS from *E. coli* (Invivogen) was administered i.p. 24 hours before the second stimulus and 90 minutes after, blood was collected and centrifuged at 6000 rpm for 10 minutes to obtain the serum for detection of cytokine levels.

#### 2.8.1 Flow cytometry

Bone marrow was removed as described above and cells were restimulated *ex vivo* with LPS (10 ng/ml) or allowed to rest for 6 days and then restimulated with LPS (10 ng/ml) or PolyIC (100 µg/ml), as described above (section 2.8). After 1 h of LPS or PolyIC stimulation, cells were treated with GolgiPlug, and four hours later, the cells were stained for surface marker, and then fixed, permeabilized and intracellularly stained for cytokines following the manufacturer’s instructions. Cells were stained with antibodies against CD11b, Ly6G, Ly6C, CD49b, TNF and IL-6 (BD Pharmingen and Biolegend).

Flow cytometry data were acquired on a FACS Canto II cytometer (BD Biosciences). Data was analyzed using FACS Diva software and FlowJo10.10 (BD Biosciences). High dimensional data analysis was performed using tSNE for dimensionality reduction, X-Shift for clustering, Cluster Explorer and Euclid for clustering quality control.

### 2.9 Tumor models and immunization

For the prophylactic approach in mice, 1 x 10^6^ CFU of *Salmonella* LVR01 were i.p administered one week or one month before tumor implantation.

Tumor implantation: B16F10 and A20 cell lines were grown in culture and harvested in log phase. Then, cells were washed and resuspended in saline, resulting in final concentrations of 2.5 x 10^6^ cells/ml or 5 x 10^6^ cells/ml, respectively. Mice were injected subcutaneously (s.c.) into the right flank with 0.1 ml or 0.2 ml, respectively. Tumor measurements were taken every other day with a microcaliper, and tumor volumes were calculated using the formula length x width x depth x π/6. Euthanasia was carried out by cervical dislocation when tumors reached 2000 mm^3^ or earlier if animals displayed signs of distress.

#### 2.9.1 Flow cytometry

On day 14 or 17 post tumor implantation, mice were sacrificed and tumors were removed. Single cell suspensions were obtained by mechanical disaggregation. Cells were stained with antibodies against CD45, CD11b, NK1.1 or CD49b and CD8 (BD Pharmingen and Biolegend).

Mice were sacrificed at day 3, 6, 9, 22 and 27 post LVR01-treatment; blood, bone marrow and tumor tissue when available (at days 22 and 27) were removed. Red blood cells were then lysed using the Qiagen Erythrocyte Lysis Buffer (Cat No 79217). Following the manufacturer’s suggestions, cells were prepared as described above and stained with antibodies against CD45, CD11b, Ly6G, CD49b and F4/80 (BD Pharmingen and Biolegend).

Data acquisition and analysis were performed as described above.

#### 2.9.2 Gene expression analysis

In independent experiments, mice were sacrificed at day 14 post-tumor implantation, and tumors were removed, collected in TRizol reagent (Invitrogen) and stored at -80°C until processed as previously described (19). Following retrotranscription, quantitative RT-PCR for *Tnf, Il6, Ifng, Il12, Cxcl1, Cxcl2, Cxcl9, Cxcl10, Il1a, Il1b, Ccl2, Ccl3, Ccl4, Ccl5, Ccl20* and *Tlr4* mRNA were conducted using QuantiTect^®^ SYBR^®^ Green PCR Kit (Qiagen) in a Rotor-Gene 6000 (Corbett), primer sequences are available under request. This panel was based on our previous work with modifications (15). β-actin encoding gene was used as the housekeeping gene. The relative mRNA amount in each sample was calculated using the 2^−ΔΔCt^ method as previously described (20) where ΔCt = Ct_gene of interest_ − Ct_housekeeping_, and expressed as relative mRNA levels in the test groups compared to the control group.

### 2.10 ELISA

Cytokine production was measured in cell culture supernatants or mouse sera using commercial ELISA kits for humans or mice (R&D Systems and Biolegend, respectively), following the manufacturer’s instructions.

### 2.11 Lactate assay

Lactate concentrations were measured in cell culture supernatants on days 1 and 6 of the training protocol using a coupled enzymatic assay in which lactate was oxidized. The resulting H_2_O_2_ was coupled to the conversion of Amplex Red (Life Technologies) reagent to fluorescent resorufin by HRP (horseradish peroxidase, Thermo Scientific) (21).

### 2.12 Statistical analysis

Differences in survival times were determined using Kaplan–Meier and log-rank tests using GraphPad Prism 8 software. The statistical significance of differences between study groups was analyzed using the Student T-test. In every case, p < 0.05 was considered statistically significant.

## 3 Results

### 3.1 *Salmonella* induces trained immunity-like response in whole bone marrow cells but immune tolerance in bone marrow-derived myeloid-enriched cells

**Figure 1:**
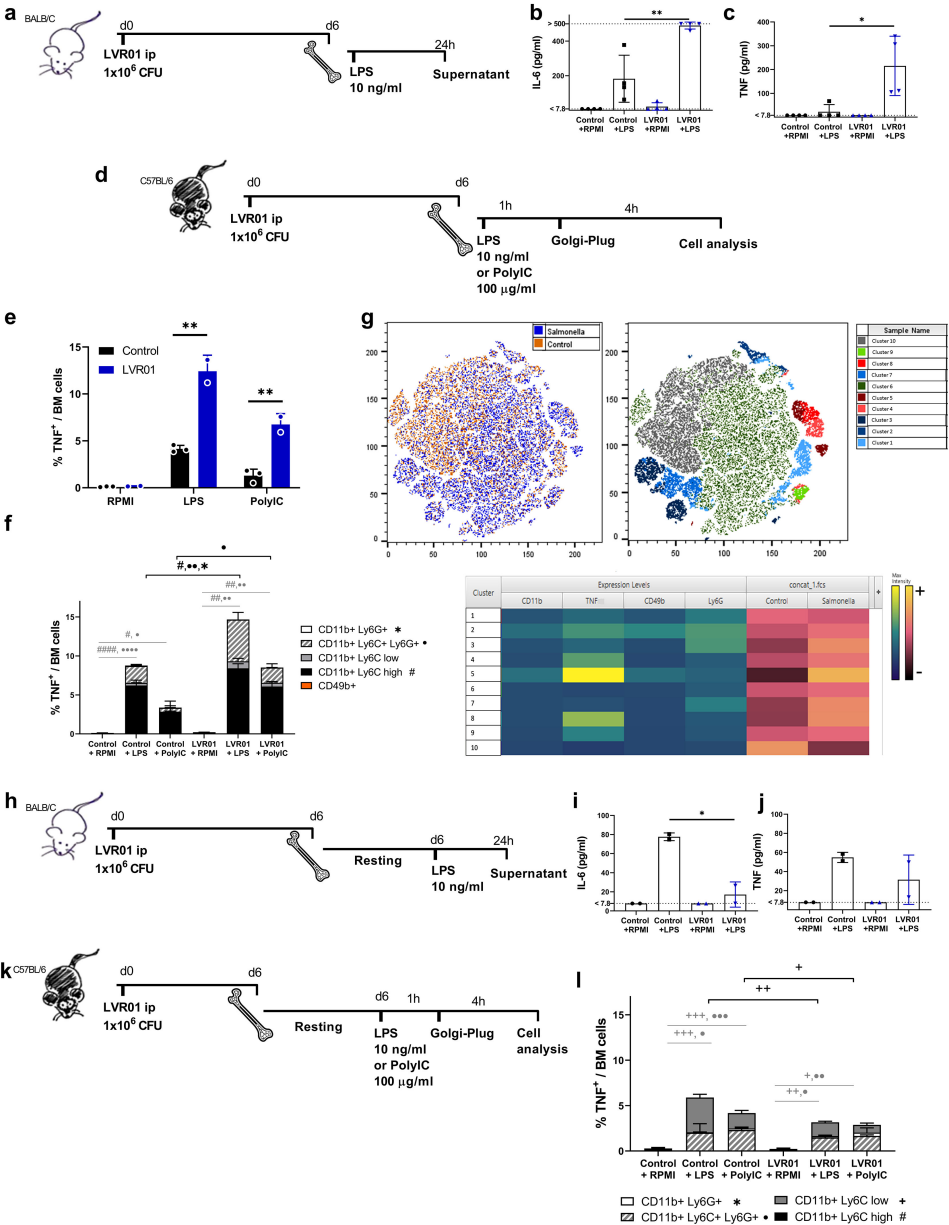
*Salmonella* increases pro-inflammatory cytokines in bone marrow cells upon *ex vivo* restimulation. **(a,d,h and k)** Experimental setups, “d” for days and “h” for hours. **(b)** IL-6 and **(c)** TNF release from bone marrow cells trained with PBS or LVR01 (1 x 10^6^ CFU) *in vivo* after LPS (10 ng/ml) restimulation *ex vivo* at day 6, measured by ELISA. Flow cytometry analysis of **(e)** total TNF+ and **(f)** cell populations producing TNF within bone marrow cells from trained-mice, *ex vivo* restimulated with LPS or Poly I:C at day 6 and then treated with GolgiPlug. **(g)** Unsupervised analysis of single-cell events from the flow cytometry data of bone marrow using the t-SNE algorithm. The t-SNE plot of concatenated BM Control+LPS and BM LVR01+LPS is shown. The heatmap displays the expression levels of the selected markers on the concatenated t-SNE plots. **(i)** IL-6 and **(j)** TNF release from bone marrow cells from LVR-trained mice and control ones (PBS), allowed to rest for 6 days before *ex vivo* restimulation with LPS, measured by ELISA. **(l)** Flow cytometry analysis of TNF-producing cell populations within bone marrow cells from LVR- trained mice and control ones (PBS), allowed to rest before *ex vivo* restimulation with LPS and then treated with GolgiPlug. Data are presented as mean ± SD (*p < 0.05, **p < 0.01, ***p < 0.001 by t test).

To evaluate the capability of *Salmonella* LVR01 to promote a stronger response upon a second stimulus, mice underwent pre-treatment with *Salmonella* or PBS as control, and after 6 days, bone marrow cells were extracted. The entire bone marrow samples were subsequently restimulated *ex vivo* with LPS. Following a 24-hour incubation period, levels of IL-6 and TNF were measured in supernatants. Both cytokines were increased in the LVR01-treated group in comparison with the PBS-control group (**Figures 1a–c**). Additionally, after 4 hours of stimulation, cells were treated with GolgiPlug for intracellular staining to ascertain which bone marrow cells were responsible for the production of these cytokines (**Figure 1d**). In agreement with cytokine concentration, we found that LVR01-trained mice presented a greater percentage of TNF-producing cells in BM than in unprimed mice upon a second stimulus (**Figure 1e**). Given the important role of neutrophils and NK cells in the anti-tumoral effect of *Salmonella* LVR01 (15) and that both cell types can be trained, we investigated whether these cells were responsible for the production of proinflammatory cytokines and found that TNF-producing cells were not NK cells (**Figure 1f**). However, we found that the source of TNF was CD11b^+^ myeloid cells, mainly double-positive Ly6C^+^ Ly6G^+^ population (probably neutrophils, and not myeloid-derived suppressor cells (MDSC), due to its potential to produce pro-inflammatory cytokines), and Ly6C high monocytes. A similar scenario was observed upon a different second stimulus, i.e. Poly I:C (**Figures 1e, f**). We next used t-distributed stochastic neighbor embedding (tSNE) to perform an unsupervised analysis of the flow cytometry dataset generated from different conditions (Control+LPS and LVR01+LPS). Single-cell events were exported, concatenated, and displayed in a single tSNE heatmap plot for each condition. Heatmap representations of the expression levels of different markers defined clusters of cells (**Figure 1g**). A comparison of the different conditions showed that all cells producing TNF in the bone marrow are myeloid cell types that express CD11b markers. Intracellular IL-6 production could not be detected under these conditions.

However, when bone marrow cells were allowed to adhere (and non-adherent cells were removed) to obtained a myeloid-enriched cell population (**Supplementary Figure 1**), and then to rest in culture for 6 days prior to restimulation (**Figure 1h**), cells from the LVR01-treated group exhibited a reduction in IL-6 production upon restimulation, reaching levels comparable to those of the control group (**Figures 1i, j**). This suggests that the initially enhanced cytokine production was not sustained by these myeloid-enriched cells, but instead became tolerized in *Salmonella*-treated animals. In line with this, we observed a marked decrease in TNF production, which correlated with the disappearance of TNF-expressing Ly6C high monocytes—further supporting their central role in the proinflammatory response (**Figures 1k, l**).

### 3.2 *Salmonella* increases concentrations of pro-inflammatory cytokines in serum upon *in vivo* restimulation with LPS

**Figure 2:**
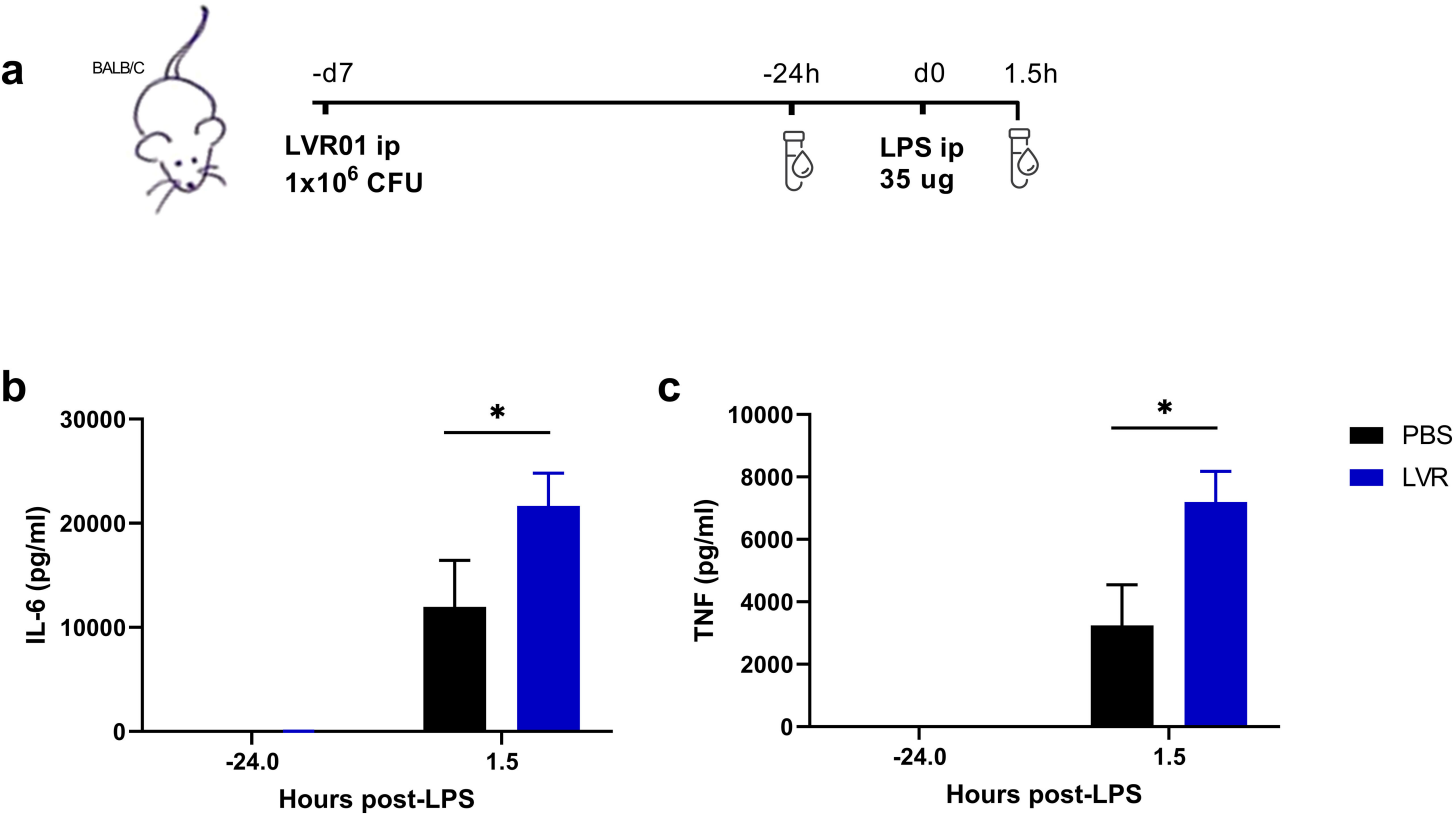
*Salmonella* increases pro-inflammatory cytokines in serum upon *in vivo* restimulation. **(a)** Experimental setup, “d” for days and “h” for hours. **(b)** IL-6 and **(c)** TNF production levels from serum of mice trained with PBS or LVR01 (1 x 10^6^ CFU) *in vivo* after LPS (35 ug) restimulation at day 7, measured 24 hours before and 90 minutes after LPS challenge, measured by ELISA. Data are presented as mean ± SD (*p < 0.05 by t test).

To study the potential of *Salmonella* in inducing trained immunity *in vivo*, mice were treated with either LVR01 or PBS. After 7 days, they were restimulated with LPS i.p. Blood samples were collected 24 hours before and 90 minutes after LPS administration (**Figure 2a**). Mice pre-treated with LVR01 exhibited increased levels of IL-6 and TNF compared to the control group (**Figures 2b, c**).

### 3.3 *Salmonella* induces immune tolerance in human adherent monocytes *in vitro*

**Figure 3:**
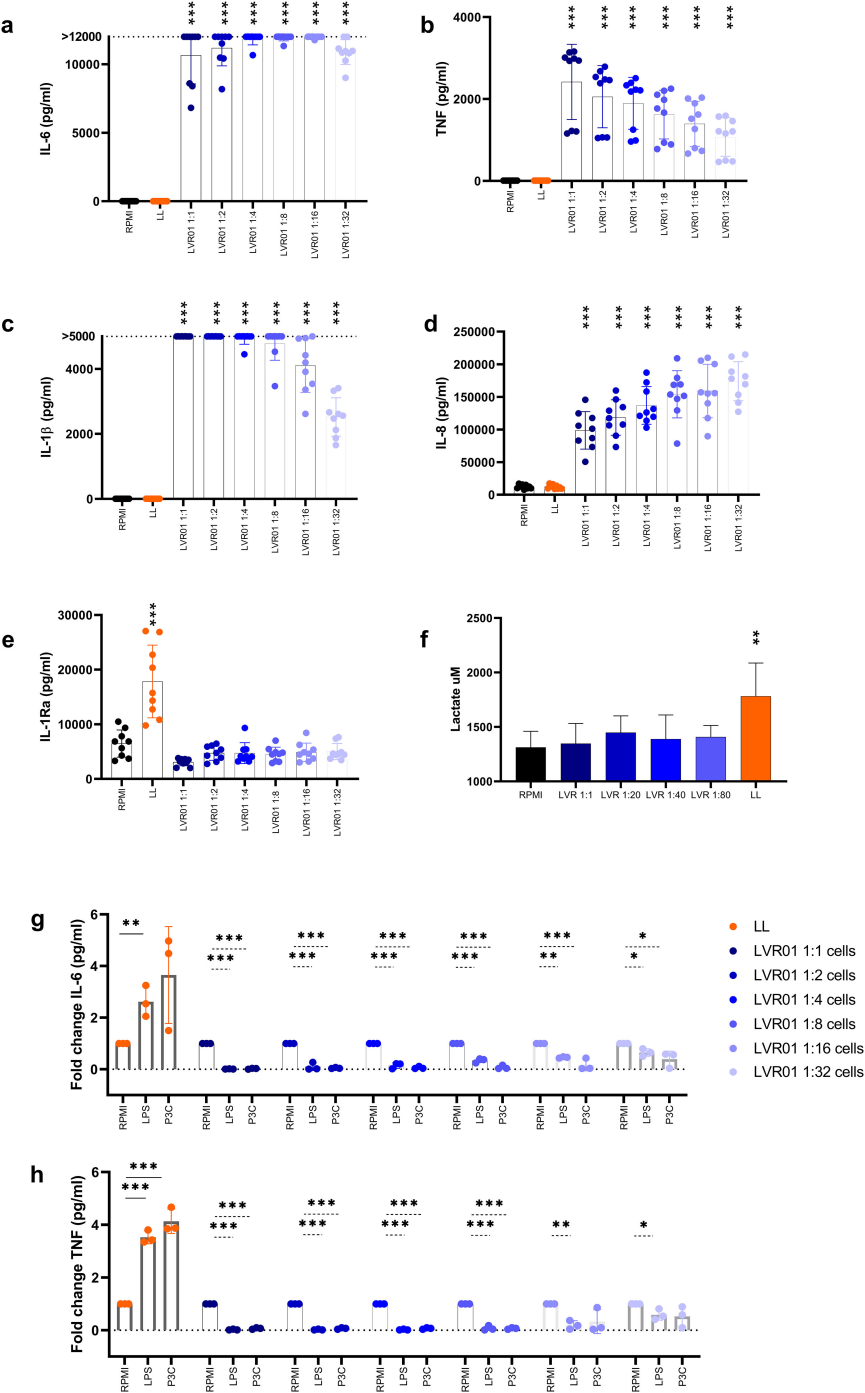
*Salmonella* induces immune tolerance *in vitro*. **(a)** IL-6, **(b)** TNF, **(c)** IL-1β, **(d)** IL-8 and **(e)** IL-1Ra release from monocytes-trained with RPMI, *L. braziliensis* lysates (25 µg/ml) and different MOI of *Salmonella* LVR01:cells (1:1, 1:2, 1:4, 1:8, 1:16, 1:32) for 1 hour, and measured after 24 hours by ELISA. **(f)** Lactate production assessed in the supernatants of trained monocytes upon second stimulus by fluorometric assay. Data are presented as mean ± SD (*p < 0.05, **p < 0.01, ***p < 0.001 by t test) **(g)** IL-6 and **(h)** TNF release from monocytes-trained with RPMI, *L. braziliensis* lysates and different MOI of *Salmonella* LVR01:cells (1:1, 1:2, 1:4, 1:8, 1:16, 1:32) for 1 hour, and restimulated with LPS (10 ng/ml) or Pam_3_Cys (10 µg/ml) at day 7, measured by ELISA. Data are represented as fold increase normalized to RPMI (non-trained cells) and shown as mean ± SD (*p < 0.05, **p < 0.01, ***p < 0.001 by t test).

To investigate the potential of *Salmonella* LVR01 to induce trained immunity *in vitro*, human adherent monocytes were stimulated by infection with varying multiplicities of infection (MOI) of *Salmonella* for one hour, and then gentamicin was added. In addition, monocytes were stimulated with *Leishmania braziliensis* lysates (LL) or remained without stimuli (RPMI). Cytokine levels were assessed in the supernatant 24 hours after the initial stimulus. Significantly elevated concentrations of IL-6, TNF, IL-1β, and IL-8 (in a MOI-dependent manner) were observed in cells infected with *Salmonella* compared to the RPMI and LL groups (**Figures 3a–d**). Instead, IL-1Ra production was heightened in the LL group compared to the other experimental groups (**Figure 3e**).

Alternatively, cells were allowed to rest for six days after the initial stimulus, and then they were restimulated with LPS or Pam_3_Cys for 24 hours, and proinflammatory cytokines in the supernatant were quantified. LL served as a positive control for trained immunity induction, while RPMI medium was a negative control. Despite the great initial cytokine response induced by *Salmonella*, a reduction in the production of IL-6 and TNF was observed upon restimulation (**Figures 3g, h**, **Supplementary Figure 2A**), confirming the tolerant effect induced by *Salmonella* in mouse monocytes. Additionally, lactate production was measured upon the second stimulus. Cells treated with LL maintained high lactate levels, whereas in *Salmonella*-treated cells lactate production is similar to RPMI non-trained cells (**Figure 3f**).

Next, we conducted the same trained immunity *in vitro* experiments using heat-killed *Salmonella* instead of live attenuated bacteria. Consistently, we observed the induction of human monocyte tolerance (**Supplementary Figure 2B**). The same effect was corroborated in mice, when bone marrow-derived murine cells were trained *in vitro* with LL or heat-killed LVR01, and 6 days later, myeloid-enriched cells were restimulated with LPS or PolyIC for 24 hours. The results revealed a reduction in proinflammatory cytokines in cells treated with heat-killed LVR01, as observed with human monocytes (**Supplementary Figure 2C**).

### 3.4 Pre-treatment with LVR01 inhibits tumor growth and extends animal survival

**Figure 4:**
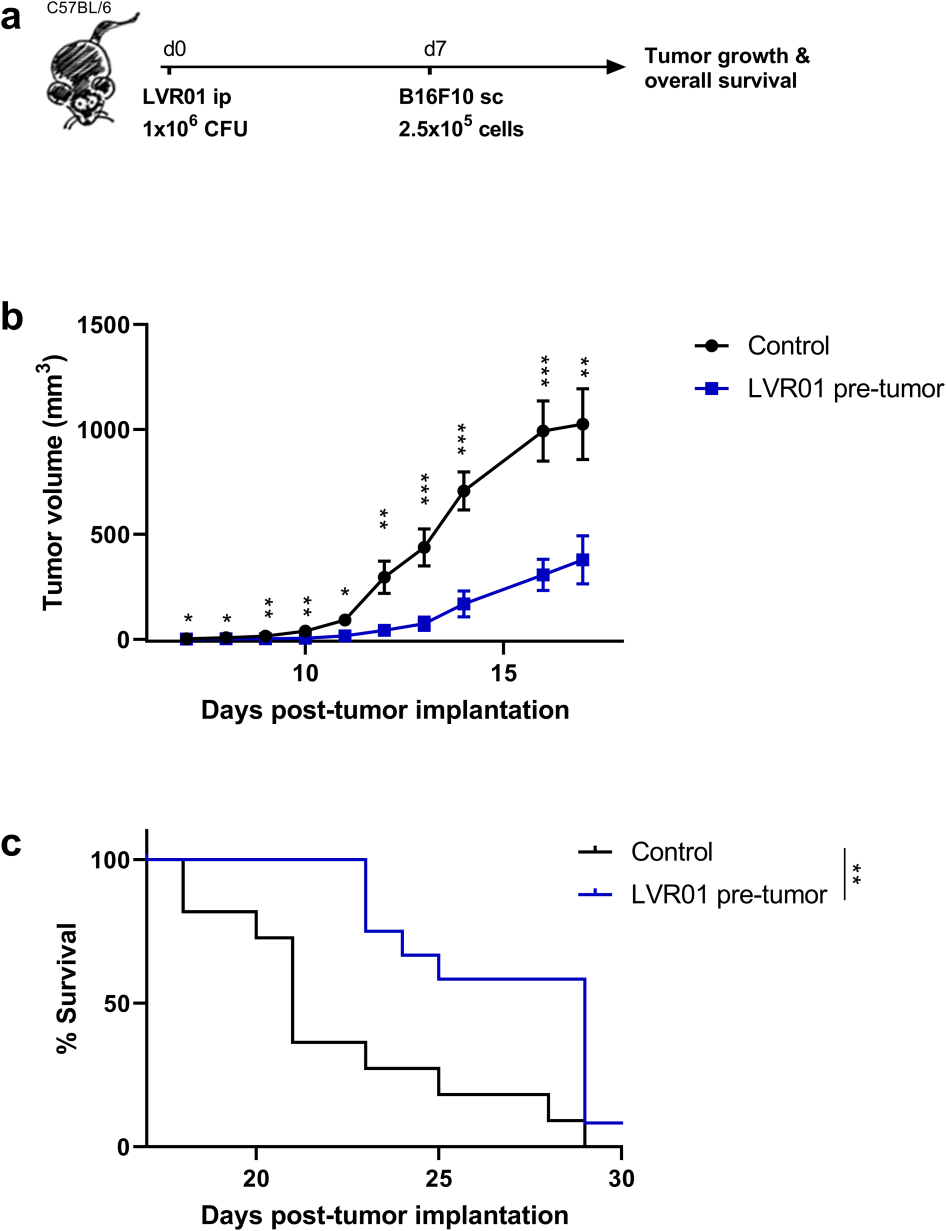
Pre-treatment with *Salmonella* inhibits tumor growth and prolongs survival of B16F10-bearing mice. **(a)** Experimental setup, “d” for days. *In vivo* **(b)** tumor growth and **(c)** survival curves of C57BL/6 mice were inoculated with 1 x 10^6^ CFU of *Salmonella* LVR01 or PBS intraperitoneally one week before B16F10 (2.5 x 10^5^ cells) implantation. Overall survival was followed up for 35 days (n=12). Significance was calculated between the groups (*p < 0.05, ** p < 0.01, *** p < 0.01 by t test and log rank, respectively).

Given the differing results from whole bone marrow cells and monocytes, we consider that the *in vivo* approach is the most comprehensive model for further investigation. Hence, we evaluated the effect of LVR01 pre-treatment on the development of a melanoma tumor implanted one week later (**Figure 4a**). Significantly reduced tumor volume was observed in mice receiving *Salmonella* compared to control mice (**Figure 4b**), a trend reflected in the overall survival of the animals (**Figure 4c**). Additionally, we examined the effects of pre-treatment with *Salmonella* one month before tumor implantation, which resulted in similar outcomes (**Supplementary Figure 3a, b**). Subsequently, we repeated the one-week pre-treatment protocol in a non-Hodgkin lymphoma model. As observed in melanoma-bearing mice, LVR01-treated mice exhibited a delay in A20-tumor growth and prolonged overall survival too (**Supplementary Figure 3c-e**).

### 3.5 LVR01 pre-treatment anti-tumor effect is not the result of an increased inflammatory response in the tumor microenvironment

**Figure 5:**
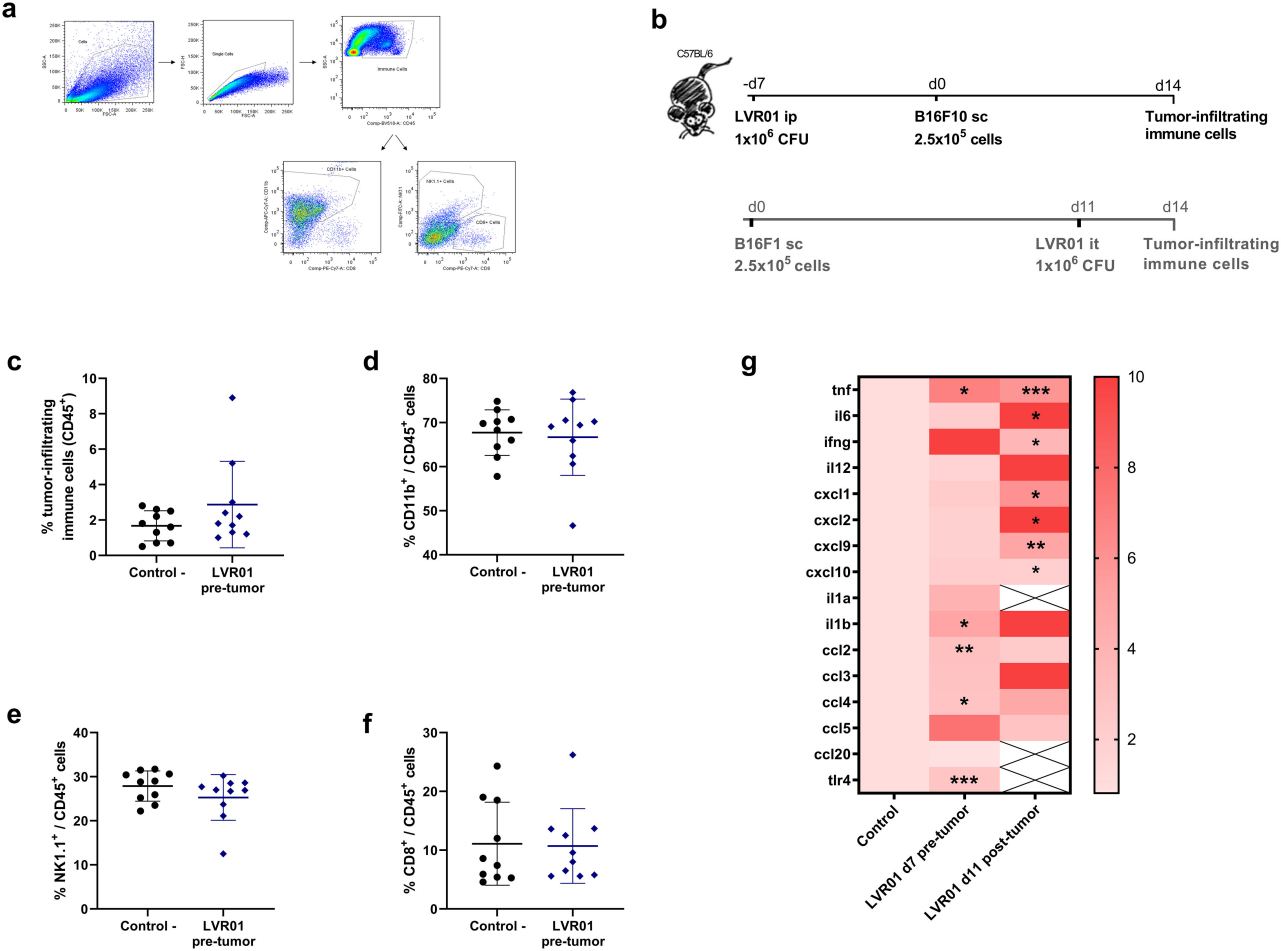
TME in mice pre-treated with *Salmonella*. **(a)** Representative flow cytometry plots and **(b)** experiment setups, “d” for days. Percentage of CD45+ **(c)**, CD45+CD11b+ **(d)**, CD45+NK1.1+ **(e)** and CD45+CD8+ **(f)** cells in the tumor microenvironment, 14 days after tumor implantation, assessed by flow cytometry in mice pre-treated with LVR01 one week before tumor implantation (n=10). **(g)** Heatmap representing cytokine/chemokine profile in *Salmonella* pre-treated (LVR01 pre-tumor, 7 days before tumor implantation) and treated (LVR01 it, 11 days after tumor implantation) mice, normalized against control untreated mice, expressed as mean Log2. Significance was calculated between the groups (*p < 0.05, ** p < 0.01, *** p < 0.01 by t test).

*Salmonella* LVR01 has been extensively studied as cancer therapeutic approach. Its anti-tumor effect is due to a strong inflammatory response in the tumor microenvironment (TME) characterized by a great increase in pro-inflammatory cytokines and chemokines, accompanied by the infiltration of immune cells, primary neutrophils, macrophages, and NK cells (13, 15). To explore whether the hereby observed prophylactic anti-tumor effect was also related to a local inflammatory immune response, we assessed the recruitment of immune cells into the tumor. When melanoma tumors reached a reasonable size and can be excised (approx. 14 days after implantation, see M&M section), the percentage of tumor-infiltrating immune cells was assessed. The results revealed no discernible difference in the percentage of CD45+, CD11b+, NK1.1+, or CD8+ tumor-infiltrating immune cells between LVR01 and control group (**Figure 5**). Moreover, when we analyze the transcriptional profile of the TME 14 days after tumor implantation in the prophylactic model (LVR01 pre-tumor) and in the therapeutic model (LVR01 intratumoral), we observed differences in the expression of pro-inflammatory cytokines and chemokines. Although pre-treatment with *Salmonella* induces mRNA expression of certain cytokines, the pro-inflammatory signature in the TME is not so noticeable as in LVR01 intratumoral treatment (**Figure 5g**). Furthermore, similar outcomes were observed in the A20 non-Hodgkin lymphomas, 17 days after tumor implantation (**Supplementary Figure 4**).

### 3.6 Kinetics of immune effector cells over time after *Salmonella* administration

**Figure 6:**
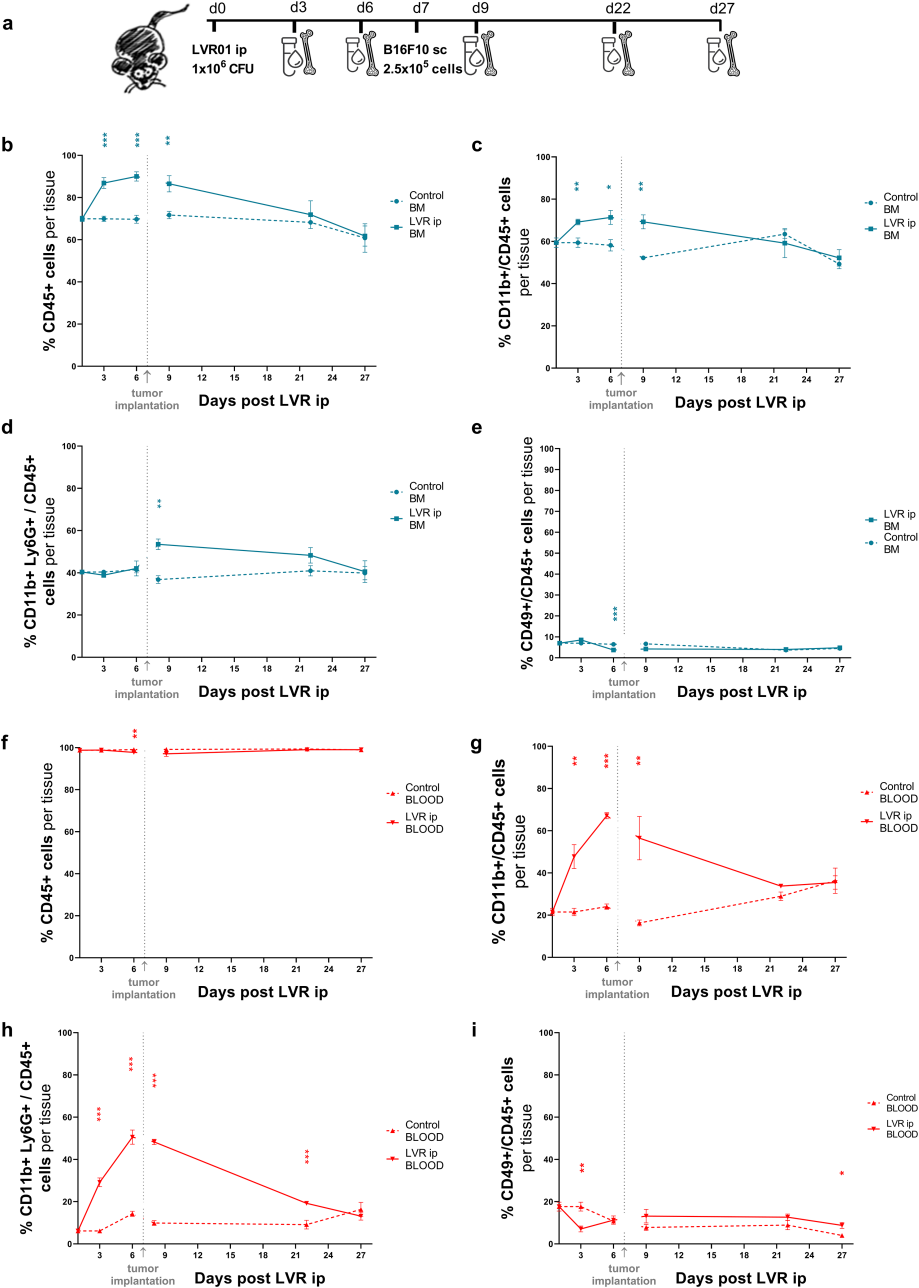
Kinetics of immune effector cells after *Salmonella* administration. **(a)** Experimental setup, “d” for days. Kinetics of CD45+ **(b)**, CD45+CD11b+ **(c)**, CD45+CD11b+Ly6G+ **(d)** and CD45+CD49b+ **(e)** cells in bone marrow samples from mice pre-treated with LVR01 one week before B16F10 tumor implantation. Kinetics of CD45+ **(f)**, CD45+CD11b+ **(g)**, CD45+CD11b+Ly6G+ **(h)** and CD45+CD49b+ **(i)** cells in blood samples from mice pre-treated with LVR01 one week before B16F10 tumor implantation. Data are presented as mean ± SEM (*p < 0.05, **p < 0.01, ***p < 0.001 by t test).

To evaluate the role of immune effector cells in the prophylactic anti-tumor effect of *Salmonella* LVR01, we studied the kinetics of specific cell populations over time. Mice were treated with LVR01 or PBS intraperitoneally, and one week later, B16F10 melanoma cells were implanted subcutaneously. Blood and bone marrow samples were collected on days 3, 6, 9, 22 and 27 post-LVR01 treatment (**Figure 6a**). Animals treated with *Salmonella* rapidly exhibited an increased percentage of CD45+ and specifically CD45+CD11b+ myeloid cells in bone marrow (**Figures 6b, c**). In blood, the percentage of CD45+ cells remained unchanged during the experiment (**Figure 6f**), while CD45+CD11b+ myeloid cells markedly increased after LVR01 treatment (**Figure 6g**). In particular, there is an increased percentage of CD45+CD11b+Ly6G+ cell population in the blood post-LVR01 treatment, which is sustained for days and then decreases over time (**Figure 6h**). This cell population also increased in bone marrow, but only after tumor implantation (**Figure 6d**). No significant differences were observed between the percentage of CD45+CD49b+ cells, neither in bone marrow or blood (**Figures 6e, i**). We used t-distributed stochastic neighbor embedding (tSNE) to perform an unsupervised analysis of the flow cytometry dataset generated from different conditions (Control and *Salmonella*) at different bone marrow and blood time points. Single-cell events were exported, concatenated, and displayed in a single tSNE heatmap plot for each condition. Heatmap representations of the expression levels of different markers defined clusters of cells (**Supplementary Figure 5**). Additionally, we studied the percentage of tumor-infiltrating immune cells at days 22 and 27 post-LVR01, and the results revealed no discernible difference between the LVR01 and control groups (data not shown).

## 4 Discussion

In this study, we investigated the innate immune response induced by *Salmonella* and its effect on subsequently implanted tumors.

First, we demonstrate that mice pre-treated with LVR01 have a significant increased production of pro-inflammatory cytokines by bone marrow cells upon exposure to LPS. Additionally, our study identified that the primary producers of TNF were cells derived from the myeloid lineage (**Figure 1**). These results suggest that LVR01 modifies the central immune response to a secondary stimulus, resembling the trained immunity response (22). However, after allowing cell cultures to rest for 6 days and then exposing the adherent monocyte-enriched cell population to LPS, these cells exhibited a tolerant response (**Figure 1**). A dose-dependent tolerogenic effect was observed when priming human monocytes with different MOI of LVR01 (**Figure 3**). It is well known that large amounts of LPS induce immune paralysis or LPS tolerance in monocytes (23). Some mechanisms underlying the LPS-tolerant effect involve metabolic reprogramming and epigenetic modifications characterized by selective and transient silencing of pro-inflammatory genes (2). LPS triggers the production of itaconate and prevents the accumulation of fumarate, a crucial metabolite for the induction of trained immunity. As a consequence, these cells maintain stable lactate levels, whereas trained cells exhibit increased lactate production (1). We observed this behavior in our *in vitro* studies using human adherent monocytes. We found that LVR01 strongly induced monocytes to produce proinflammatory cytokines during the first 24 hours after stimulation. However, after a resting period, those cells developed an immune-tolerant response (**Figure 3**). Interestingly, our findings contrast with previous reports describing a biphasic, dose-dependent response to LPS stimulation, where low doses of LPS induce trained immunity, whereas high doses promote tolerance (24, 25). These studies, however, used experimental designs that differ from ours, particularly in two aspects: the studied cell population and timing of restimulation. For example, Lajqi et al. observed a dose-dependent response in a short-term experimental setup of neutrophils, where restimulation occurred within 24–48 hours. In contrast, our study focuses on a longer time frame, with bone-marrow cells resting for six days before the second stimulus. These differences in cell population and experimental conditions may be crucial in shaping the outcome of innate immune memory. In contrast, our positive control using *Leishmania* lysates induced trained immunity, as was previously reported (5), characterized by an enhancement in the production of pro-inflammatory cytokines after restimulation with LPS, and an increase in lactate production six days after the initial stimulus (**Figure 3**). It has already been reported and reviewed by Geckin et al., that it is possible to induce trained immunity in multiple types of mononuclear myeloid cells like monocytes, macrophages, dendritic cells and neutrophils, and even in lymphoid cells such as NK cells and innate lymphoid cells (22, 26–31). We hypothesized that *Salmonella* is capable of inducing central changes that are beneficial for an anti-tumor immune response, but also induces peripheral tolerance, which may explain the transient effect of the treatment in preclinical models (13, 15).

To confirm if those changes are actually triggering an anti-tumor immune response, we examined the role of LVR01 on tumor development by giving the treatment one week before tumor implantation. This experimental design is an adaptation of Kalafati et al. work, where they demonstrate that trained immunity promotes anti-tumor activity in preclinical models pre-treated with β-glucan (32). When animals were pre-treated with *Salmonella* LVR01, there was a delay in tumor growth and a prolonged overall survival (**Figure 4**). This effect was observed even when mice were pre-treated with LVR01 one month before tumor implantation (**Supplementary Figure 3**). Strikingly, the anti-tumor effect was unrelated to increased tumor immune-infiltrating cells or a pro-inflammatory tumor microenvironment in the LVR01 group, which are hallmarks of the anti-tumor effect induced by therapeutic intratumoral injection of *Salmonella* (**Figure 5**). So far, the anti-tumor effect of *Salmonella*-based cancer therapies has always been related to the bacteria’s ability to induce a strong inflammatory response in the tumor microenvironment, which is characterized by a significant increase in pro-inflammatory cytokines and chemokines, accompanied by the infiltration of immune cells, primarily neutrophils, macrophages, and NK cells (13, 15).

Kalafati et al. demonstrate that in the context of anti-tumor immunity, neutrophils and granulopoietic progenitors, not monocytes, are the major cellular effectors (32). Considering this, we continued exploring the kinetics of different immune effector cell populations in our pre-clinical model of trained immunity over time. As expected, after LVR01 treatment we observed an increase of myeloid cells in the blood and in bone marrow. This is reasonable because we are administering attenuated bacteria that cause a controlled infection in the animals. The behavior of CD11b+ Ly6G+ cells in the bone marrow caught our attention; this population significantly increased in bone marrow after tumor implantation (**Figure 6**). These results are in line with the hypothesis of how BCG-trained cells could increase cytotoxic leukocyte responses; following cancer cell death, DAMPs can be released and act as a second stimulus for trained cells, leading to an enhanced anti-tumor effect (8). It is possible that the administration of tumor cells sends a second signal that modulates the granulopoiesis in the bone marrow, reflected in an increase in the percentage of neutrophils and its progenitors (**Figure 6**). Yet, the role of neutrophils has not been demonstrated, because of major experimental limitations: (i) difficulty to deplete only neutrophils without affecting other immune cells, and (ii) impossibility to maintain neutrophil depletion over the time, due to the rapid turnover and the boost in cell numbers upon depletion. The fact that the anti-tumor effect of *Salmonella* persists even when tumors are implanted one month post-treatment suggests the induction of long-term alterations in immune cells (32).

All in all, our results show that administration of live attenuated *Salmonella* induces an immunological imprinting with a two counterposed behavior that nevertheless results in anti-tumor activity that lasts for at least one month after administration. Strikingly, this anti-tumor activity is developed in the absence of marked immune cell infiltration and inflammation in the tumor microenvironment suggesting a so far not described new mechanism for the anti-tumor effect of *Salmonella*. Digging into this new mechanism shall provide valuable information to leverage the design of new *Salmonella* mutants with improved anti-tumor effectiveness and clinical potential.

## Data Availability

The original contributions presented in the study are included in the article/**Supplementary Material**. Further inquiries can be directed to the corresponding authors.
