# Supplementary material for: Salmonella LVR01 triggers antagonistic two-armed innate immune memory that impacts on antitumor efficacy
Source: Front Immunol. 2025 Apr 30;16:1535131. doi: 10.3389/fimmu.2025.1535131 (PMC12075395; doi:10.3389/fimmu.2025.1535131)
Supplement: Supplementary file 1 [file DataSheet1.docx]

**
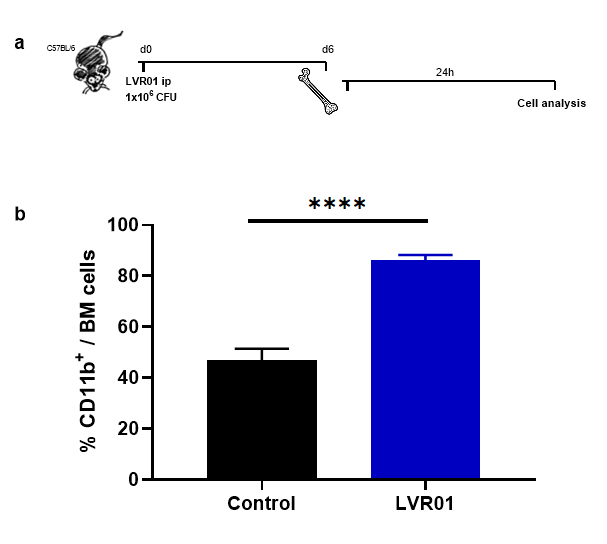
Supplementary Figure 1. Characterization of CD11b+ bone marrow-derived cells after 24 h.** (a) Experimental setup, “d” for days and “h” for hours. (b) Percentage of CD11b+ cells in bone marrow-derived cell at 24 h post-plating. Data are presented as mean ± SD (****p < 0.0001 by t test)


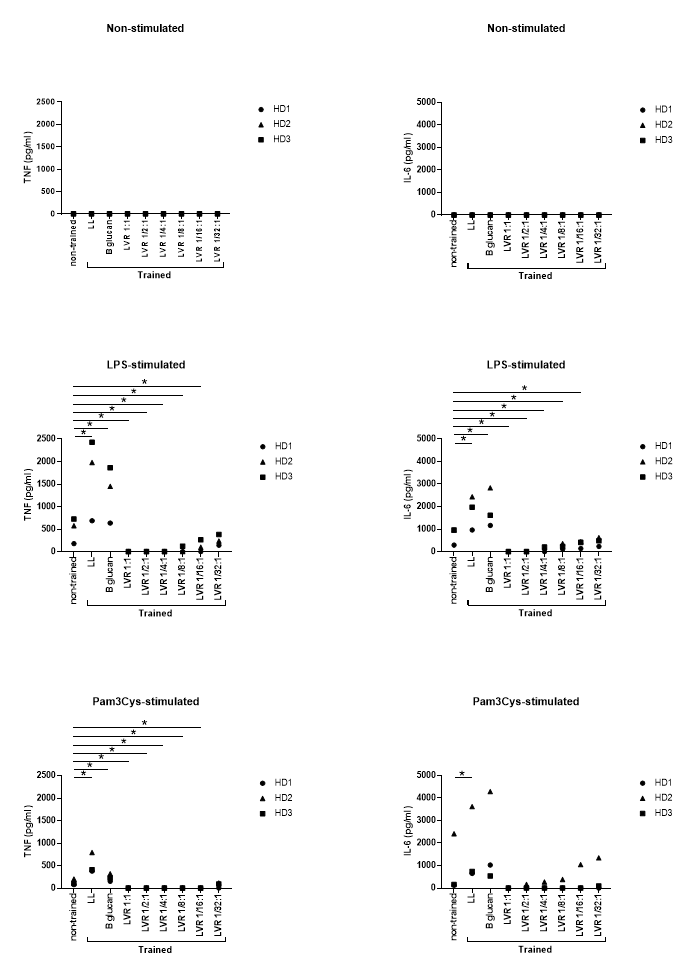
**Supplementary** **Figure 2A. *Salmonella* induces immune tolerance *in vitro*.** TNF and IL-6 release from monocytes from healthy donors (HD) trained with RPMI*, L. braziliensis* lysates (LL), β-glucan and different MOI of *Salmonella* LVR01:cells (1:1, 1:2, 1:4, 1:8, 1:16, 1:32) for 1 hour after LPS (10 ng/ml) or Pam_3_Cys (10 µg/ml) restimulation at day 7, measured by ELISA. Data are represented as concentration pg/ml and shown as individual values (*p < 0.05, **p < 0.01, ***p < 0.001 by t test).

**Supplementary Figure 2B. Heat-killed *Salmonella* induces immune tolerance *in vitro*.** (a) IL-6, (b) TNF, (c) IL-1β, (d) IL-8 release from monocytes trained with RPMI, *L. braziliensis* lysates (25 µg/ml) and different MOI of heat-killed LVR01:cells (1:1, 1:2, 1:4, 1:8, 1:16, 1:32) for 1 hour after 24 hours, measured by ELISA. Data are presented as mean ± SD (*p < 0.05, **p < 0.01, ***p < 0.001 by t test) (e) IL-6 and (f) TNF release from monocytes trained with RPMI, *L. braziliensis* lysates and different MOI of heat-killed LVR01:cells (1:1, 1:2, 1:4, 1:8, 1:16, 1:32) for 1 hour after LPS (10 ng/ml) or Pam_3_Cys (10 µg/ml) restimulation at day 7, measured by ELISA. Data are represented as fold increase normalized to RPMI (non-trained cells) and shown as mean ± SD (*p < 0.05, **p < 0.01, ***p < 0.001 by t test).


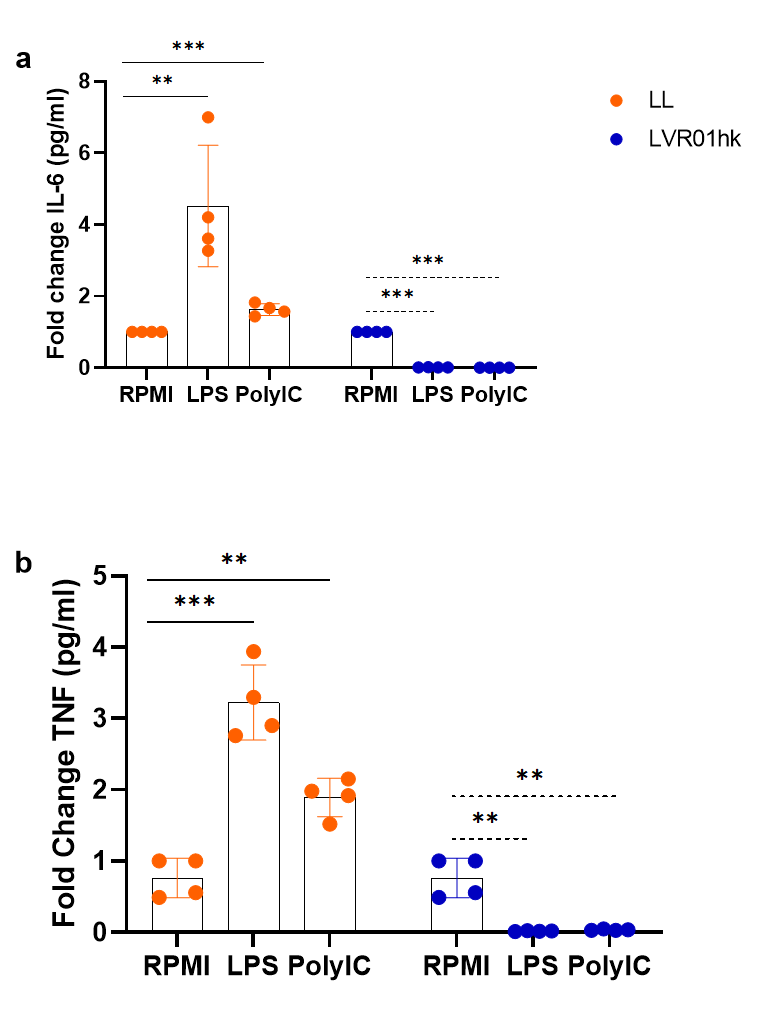


**Figure Sup 2C. Heat-killed *Salmonella* induces immune tolerance *in vitro* in mice.** (a) IL-6 and (b) TNF release from fresh bone marrow cells trained with RPMI, *L. braziliensis* lysates and 1 x 10^6^ CFU of heat-killed *Salmonella* LVR01 for 24 hours, and restimulated with LPS (10 ng/ml) or PolyIC (100 µg/ml) at day 7, measured by ELISA. Data are represented as fold increase normalized to RPMI (non-trained cells) and shown as mean ± SD (**p < 0.01, ***p < 0.001 by t test).

**Supplementary Figure 3. Pre-treatment with *Salmonella* inhibits tumor growth and prolongs survival of B16F10 and A20 non-Hodgkin-lymphoma-bearing mice.** (a and c) Experimental setups, “d” for days. *In vivo* (b) tumor growth of C57BL/6 mice were inoculated with 1 x 10^6^ CFU of *Salmonella* LVR01 or PBS intraperitoneally one month before B16F10 (2.5 x 10^5^ cells) implantation (n=12). *In vivo* (d) tumor growth and (e) survival curves of BALB/C mice were inoculated with 1 x 10^6^ CFU of *Salmonella* LVR01 or PBS intraperitoneally one week before A20 (1 x 10^6^ cells) implantation. Overall survival was followed up for 70 days (n=12). Significance was calculated between the groups (*p < 0.05, ** p < 0.01, *** p < 0.01 by t test and log rank, respectively).


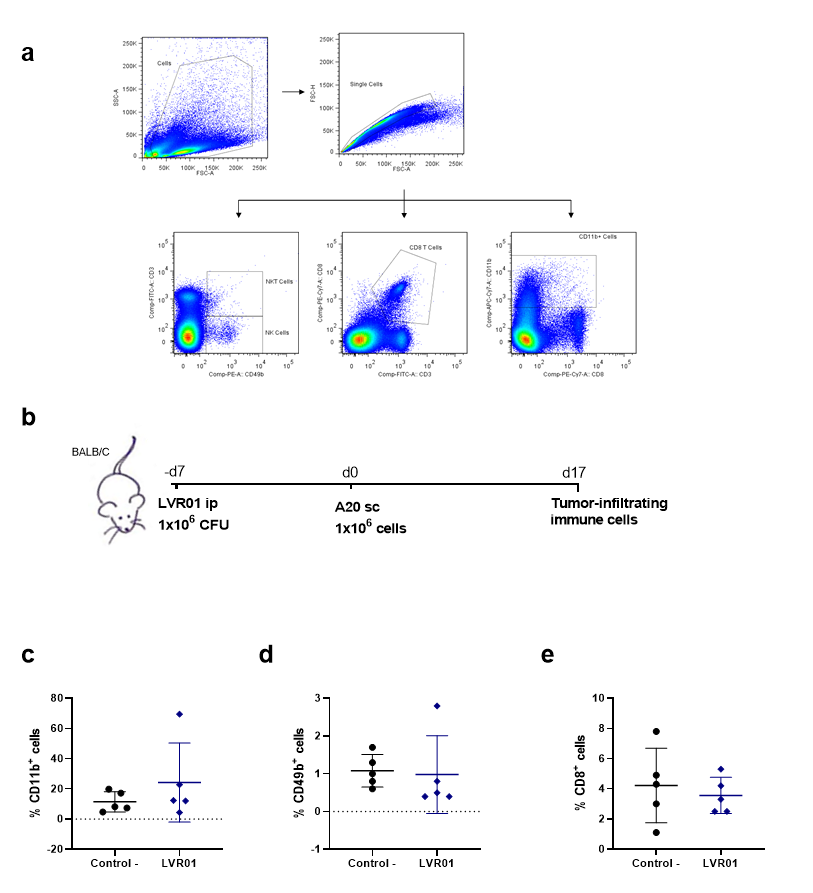


**Supplementary Figure 4. Tumor infiltrating immune cells in mice pre-treated with *Salmonella*.** (a) Representative flow cytometry plots and (b) experiment setup, “d” for days. Percentage of CD11b+ (c), CD49b+ (d) and CD8+ (e) cells in the tumor microenvironment, 17 days after tumor implantation, were assessed by flow cytometry in mice pre-treated with LVR01 one week before tumor implantation (n=5). Significance was calculated between the groups by t test.

**Supplementary Figure 5. Kinetics of immune effector cells after *Salmonella* administration.** (a) Experimental setup, “d” for days. Unsupervised analysis of single-cell events from the flow cytometry data of (b) bone marrow or (c) blood using the t-SNE algorithm. The t-SNE plot of concatenated BM Control and BM LVR01, or blood Control and blood LVR01, is shown at different time points. Heatmaps display the expression levels of the selected markers on the concatenated t-SNE plots.
